# Fatal Neonatal Cardiac Rhabdomyoma Presenting With Pulmonary Hypoplasia: An Autopsy Case Report From Jamaica

**DOI:** 10.7759/cureus.107529

**Published:** 2026-04-22

**Authors:** Scott Williams, Charles Anderson

**Affiliations:** 1 Pathology, Spanish Town Hospital, Spanish Town, JAM

**Keywords:** benign cardiac tumor, cardiac rhabdomyoma, pediatric autopsy, pediatric tumors, tuberous sclerosis complex (tsc)

## Abstract

Cardiac rhabdomyomas are the most common primary cardiac tumors in infants and are frequently associated with tuberous sclerosis complex. Although typically benign with a tendency for spontaneous regression, large lesions may result in significant morbidity and mortality. We report the case of a preterm female neonate in whom a large cardiac mass was detected on third-trimester ultrasound, accompanied by pleural effusion, lung hypoplasia, and ascites. At birth, the infant presented with severe respiratory distress, bradycardia, and hypoxia requiring ventilatory support, but despite initial stabilization, her condition rapidly deteriorated, leading to death on the third day of life. Autopsy revealed a large left ventricular mass measuring 5.0 cm × 3.5 cm × 2.8 cm, associated with pericardial effusion and marked pulmonary hypoplasia, and histopathological examination confirmed cardiac rhabdomyoma. This case highlights the potential for large cardiac rhabdomyomas to possibly contribute to fatal extracardiac complications, including pulmonary hypoplasia, particularly in resource-limited settings where advanced imaging modalities are limited.

## Introduction

Primary cardiac tumors in the pediatric population are rare, with most being benign. Among these, cardiac rhabdomyomas are the most frequently encountered tumors in neonates and infants [1,2]. Although typically asymptomatic and known to regress spontaneously, larger lesions may cause significant hemodynamic compromise, arrhythmias, or obstruction of cardiac outflow tracts [3].

Cardiac rhabdomyomas are strongly associated with tuberous sclerosis complex (TSC), with up to 80%-90% of affected patients demonstrating clinical features [4]. However, isolated cases without clear evidence of TSC have also been reported [5].

## Case presentation

A preterm female neonate was delivered at 36 weeks’ gestation via lower segment cesarean section, with a birth weight of 2,480 g. She was the second child of her mother, and there was no significant family history of congenital heart disease or genetic disorders. Antenatal ultrasound performed during the third trimester revealed a large intracardiac mass measuring 3.5 × 2.4 cm, which was associated with massive pleural effusion, lung hypoplasia, ascites, and echogenic bowel.

At birth, the neonate had APGAR scores of 7 and 9 at 1 and 5 minutes, respectively. The neonate was also noted to be bradycardic, with a heart rate of less than 60 beats per minute, and exhibited central cyanosis. Intermittent positive-pressure ventilation (IPPV) with supplemental oxygen was initiated, resulting in an improvement in heart rate above 100 beats per minute. However, the infant subsequently developed progressive respiratory distress, characterized by grunting, intercostal and subcostal retractions, and worsening hypoxemia with an SpO2 ranging between 75% and 79%. Chest radiography demonstrated hyperinflation and a markedly enlarged cardiothymic silhouette occupying much of the thoracic cavity. Despite escalation of respiratory support to nasal continuous positive airway pressure (NCPAP) and subsequently to IPPV, oxygen saturation levels continued to decline. By the second day of life, the infant developed severe hypoxemia and recurrent bradycardia, with oxygen saturation levels dropping below 20%. Despite aggressive supportive management, the clinical condition progressively deteriorated, and the neonate died on the third day of life.

Investigations

Laboratory investigations revealed leukocytosis and metabolic acidosis on arterial blood gas analysis, with serial measurements demonstrating worsening hypoxemia despite ventilatory support. Baseline complete blood count showed a hemoglobin level of 11.6 g/dL, a red blood cell count of 3.98 × 10¹²/L, a mean cell volume of 97.9 fL, a mean corpuscular hemoglobin concentration of 29.8 g/dL, a platelet count of 502 × 10⁹/L, and a packed cell volume of 38.9%. The total white blood cell count was elevated at 29.81 × 10⁹/L, with a differential count showing lymphocytes at 65.2%, neutrophils at 16.9%, and eosinophils at 0.5%.

Arterial blood gas analysis at birth demonstrated severe metabolic acidosis, with a pH of 7.01, a partial pressure of carbon dioxide (pCO₂) of 53.0 mmHg, a bicarbonate (HCO₃⁻) level of 18.2 mmol/L, a partial pressure of oxygen (pO₂) of 165 mmHg, and an arterial oxygen saturation (SaO₂) of 99.5%. Repeat analysis on day 1 showed partial correction of acidosis but marked hypoxemia, with a pH of 7.37, a pCO₂ of 31.9 mmHg, an HCO₃⁻ level of 12.7 mmol/L, a pO₂ of 41 mmHg, and a corresponding SaO₂ of 52.2%.

Renal function tests revealed a serum urea level of 4.1 mmol/L and a creatinine level of 99 µmol/L. Electrolyte analysis showed a serum sodium level of 136 mmol/L, a potassium level of 5.9 mmol/L, a chloride level of 100 mmol/L, and a bicarbonate level of 15.3 mmol/L. Liver function tests demonstrated an elevated total bilirubin level of 99.8 µmol/L, predominantly unconjugated (91.1 µmol/L), with a direct fraction of 8.7 µmol/L.Liver enzymes were also elevated, including aspartate aminotransferase at 140 U/L and gamma-glutamyl transferase at 201 U/L, while alanine aminotransferase was 29 U/L and alkaline phosphatase was 141 U/L. Serum albumin was reduced to 29 g/L.

Treatment

The neonate was managed with oxygen therapy guided by peripheral oxygen saturation monitoring. Ventilatory support included IPPV and NCPAP. A single dose of furosemide (1 mg/kg) was administered for presumed cardiac congestion. The patient remained nil per os, with gastric decompression via an orogastric tube.

Outcome

Following the infant’s death on the third day of life, a complete autopsy was performed. Gross examination revealed approximately 20.2 mL of hemorrhagic pericardial fluid and a large intracavitary mass within the left ventricle measuring 5.0 × 3.5 × 2.8 cm. The heart was markedly enlarged, and the mass exerted significant compression on the lungs, which were reduced in size and consistent with pulmonary hypoplasia.

Histopathological examination demonstrated sheets of polygonal cells with abundant glycogen-rich cytoplasm and characteristic “spider cells,” along with cytoplasmic strands radiating from centrally located nuclei. These findings were diagnostic of cardiac rhabdomyoma.

Assessment for tuberous sclerosis complex

The deceased infant had no cutaneous manifestations suggestive of tuberous sclerosis complex, and neuroimaging was not performed. The mother reported a 10-year history of depression. She reported prior pharmacologic treatment but was unable to recall the specific medication, stating that it was discontinued due to perceived lack of efficacy. She also reported associated symptoms, including insomnia and mood instability. On further dermatologic inquiry, the mother reported intermittent eczema but denied hypopigmented macules, facial angiofibromas, or other lesions suggestive of tuberous sclerosis. She has one previous child, born in 2021, now aged four years, with no history of developmental delay, dermatologic abnormalities, or other health concerns; overall development has been unremarkable. Regarding family history, the mother reported no known history of intellectual disability, malignancy, or genetic disorders on the maternal side. She noted limited knowledge of the paternal family history. The child’s father was reported to have no significant medical or psychiatric history. Genetic testing for tuberous sclerosis complex was offered but was declined by the family due to financial constraints.

Autopsy findings

**Figure 1:**
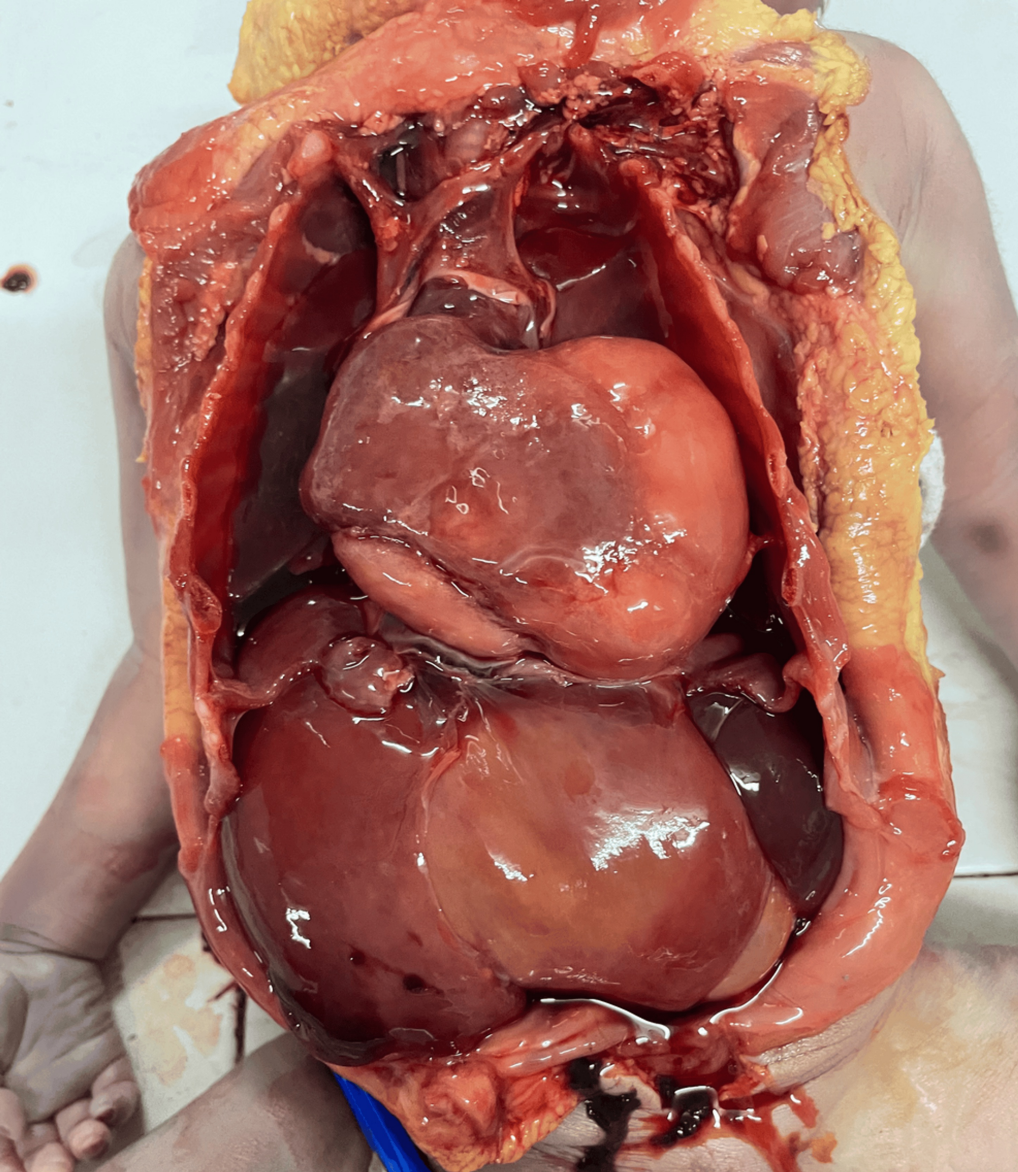
Cardiac mass in the left ventricle identified at autopsy.

**Figure 2:**
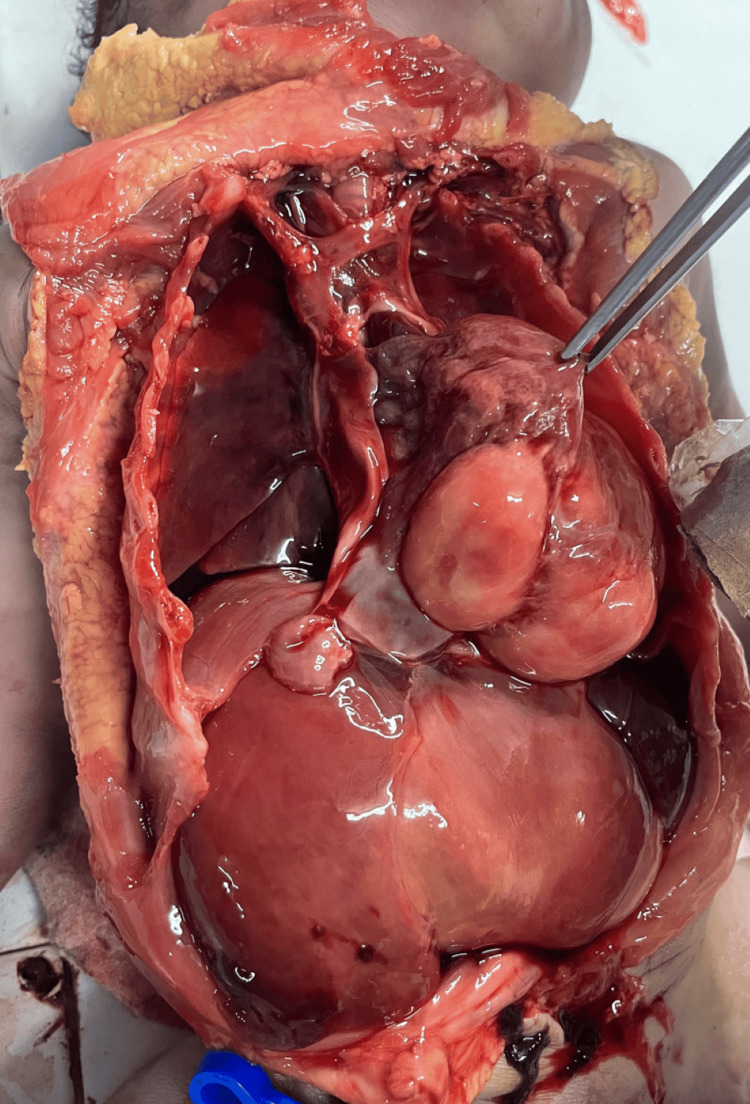
Lung hypoplasia found at autopsy.

**Figure 3:**
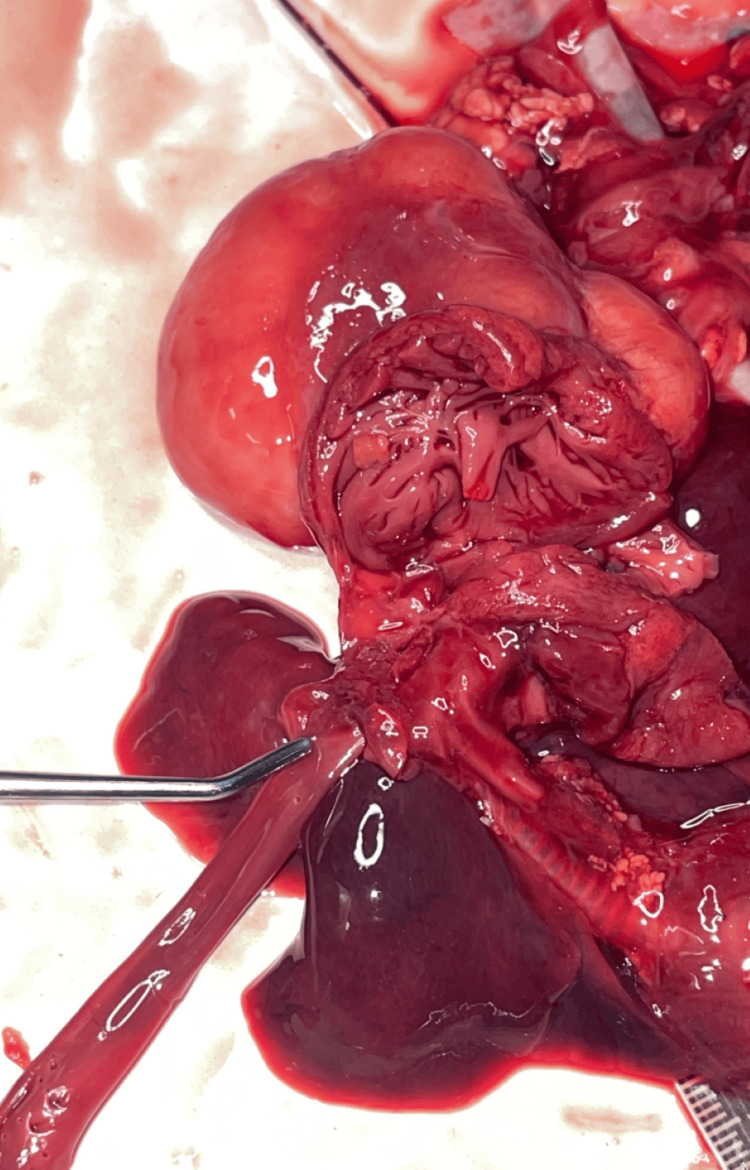
Probe within the patent ductus arteriosus.

**Figure 4:**
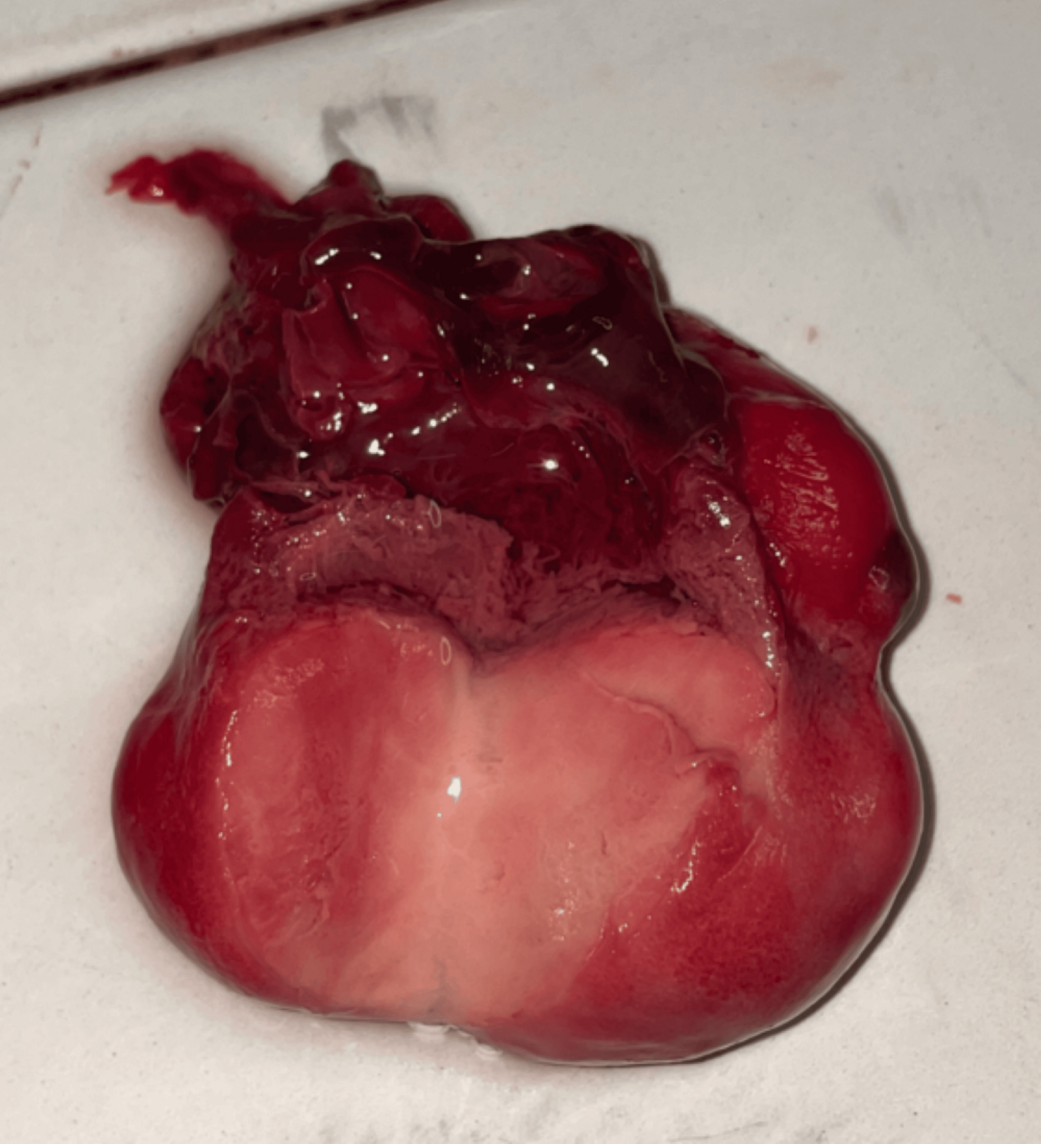
The sectioned cardiac mass.

A complete postmortem examination was performed to establish the cause of death and assess the extent of the underlying pathology. Gross examination demonstrated cardiomegaly with a large intraventricular mass occupying the left ventricle (Figure 1). The lungs were markedly reduced in size, with weights of 14.7 g for the right lung and 10.4 g for the left, and were compressed within the thoracic cavity, consistent with pulmonary hypoplasia (Figure 2). Approximately 20.2 mL of hemorrhagic pericardial fluid and a patent ductus arteriosus were identified (Figure 3). On sectioning, the left ventricular mass measured 5 × 3.5 × 2.8 cm (Figure 4). No additional major congenital anomalies were identified. These findings highlight the significant mass effect of the tumor and its contribution to both cardiac compromise and impaired pulmonary development.

Histology report

**Figure 5:**
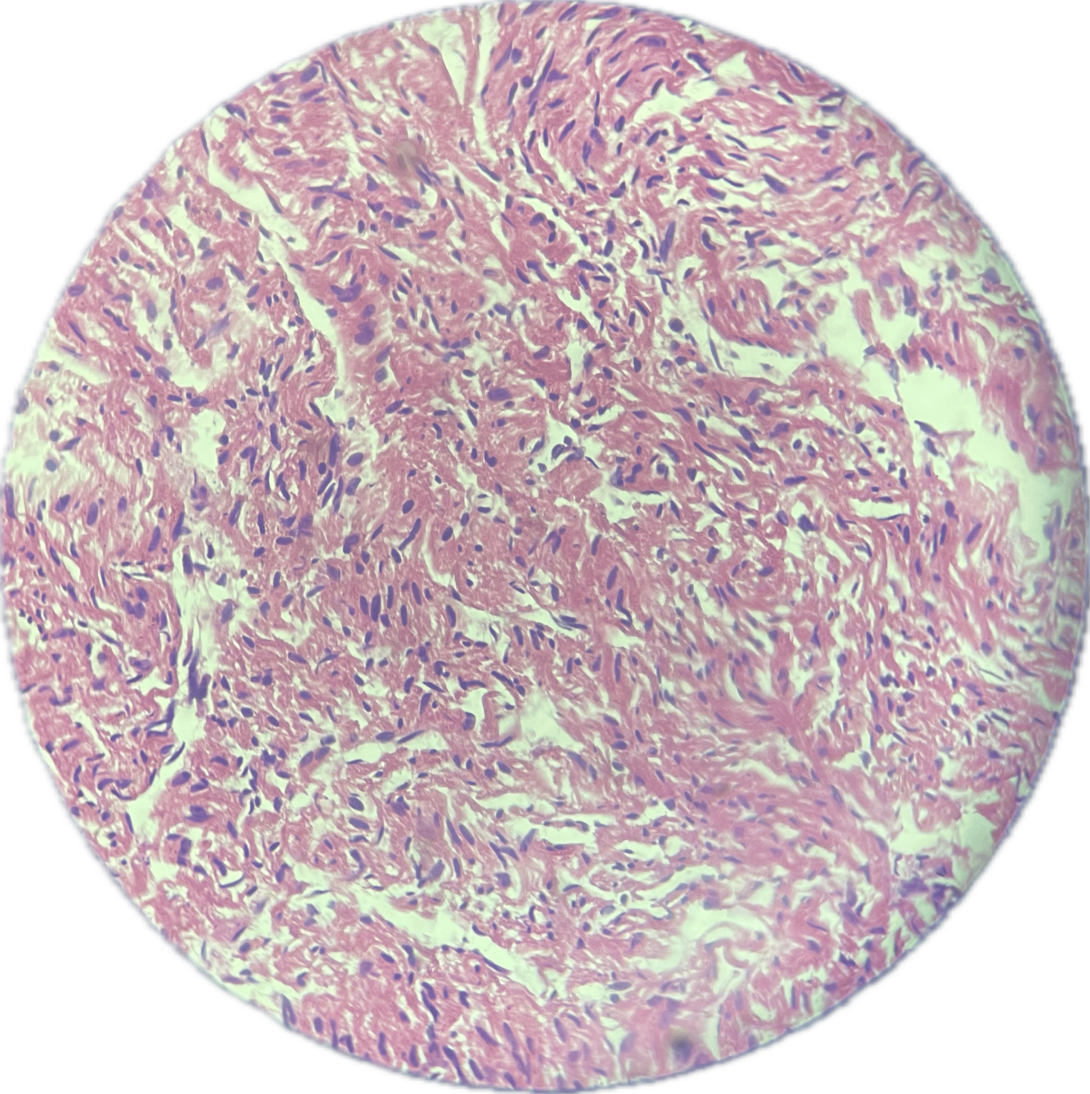
Histology of the cardiac mass showing disarray of cardiac muscle fibers.

**Figure 6:**
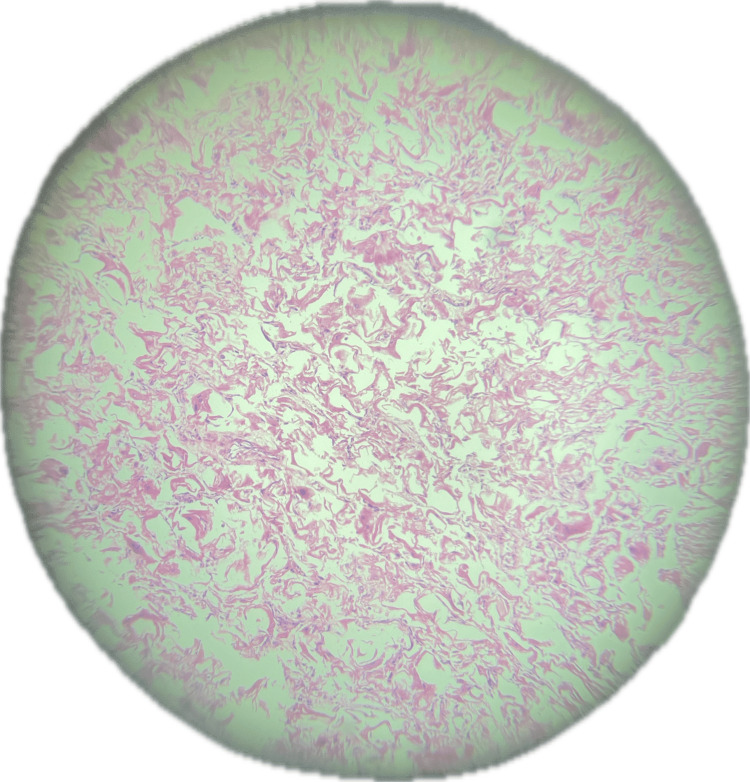
Histology of the cardiac mass showing polygonal cells ("spider cells") with glycogen vacuoles separated by cytoplasmic strands.

**Figure 7:**
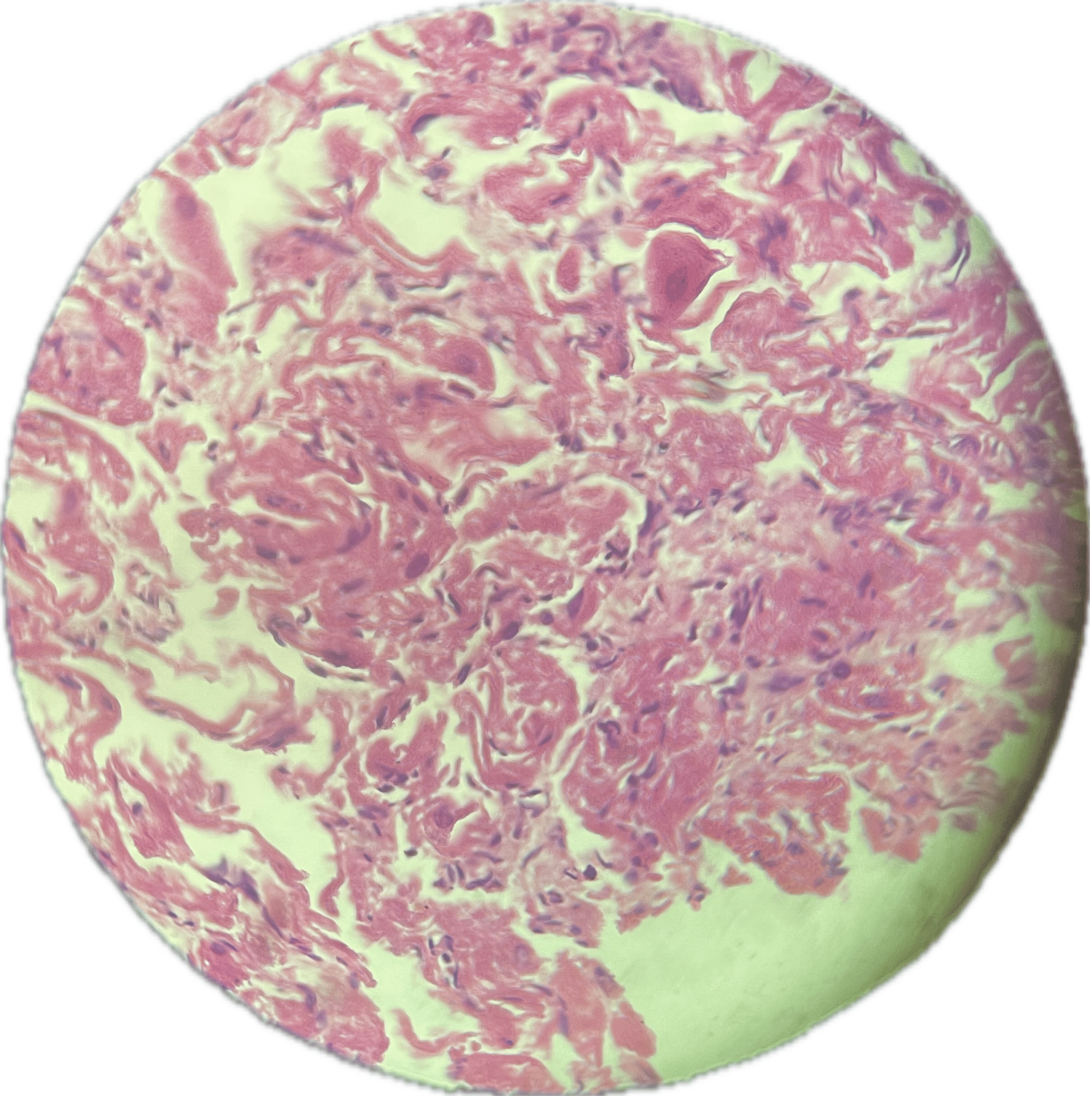
Histology of the cardiac mass showing immature cardiac muscle cells

Sections show portions of autolyzed tissue within which there is disarray of cardiac muscle fibers (Figure 5). There are also sheets of polygonal cells with abundant cytoplasm containing glycogen granules, along with markedly swollen myocytes demonstrating an almost *empty* cytoplasm with central nuclei and cytoplasmic strands, corresponding to the characteristic *spider cells* of cardiac rhabdomyoma (Figure 6). In addition, intracytoplasmic striations and immature cardiac muscle cells are identified (Figure 7). 

## Discussion

Cardiac rhabdomyomas are benign tumors of striated muscle origin and represent the most common primary cardiac tumor in infants and children [1,2]. Despite their benign histological appearance and well-documented tendency toward spontaneous regression, large lesions may result in significant morbidity and mortality due to arrhythmias, obstruction of intracardiac blood flow, or impairment of cardiac function [3,6]. The differential diagnosis of congenital intracardiac masses primarily includes cardiac fibroma, cardiac teratoma, and, less commonly, cardiac myxoma. Cardiac fibromas are typically solitary, intramural ventricular tumors composed of dense fibrous tissue and do not demonstrate spontaneous regression, distinguishing them from rhabdomyomas. Cardiac teratomas are usually pericardial or extracardiac masses arising near the great vessels and are often associated with pericardial effusion and heterogeneous tissue elements. Cardiac myxomas, although rare in neonates, are generally atrial, pedunculated lesions and occur more frequently in older patients [3,7].

The present case is notable for the unusually large size of the tumor and its possible impact on pulmonary development. Large intracardiac masses can exert significant compressive effects within the thoracic cavity, as demonstrated in this case, where the tumor likely contributed to pulmonary hypoplasia and severe respiratory compromise. This highlights the potential for even histologically benign lesions to produce life-threatening physiological consequences in the neonatal period. Pulmonary hypoplasia was further supported by morphometric assessment. Using the lung weight-to-body weight ratio (combined lung weight divided by body weight), as described in Wigglesworth's Perinatal Pathology [8], the ratio in this neonate was calculated to be 0.0115. This value falls below the accepted cutoff of 0.012, confirming pulmonary hypoplasia.

The strong association between cardiac rhabdomyomas and tuberous sclerosis complex is well established, with reported prevalence rates of up to 80%-90% [4]. However, the absence of clinical features of tuberous sclerosis in this patient underscores the occurrence of isolated rhabdomyomas. Such cases may pose diagnostic challenges, particularly in resource-limited settings where access to genetic testing is restricted [5].

Imaging modalities, particularly echocardiography and magnetic resonance imaging, play a critical role in the diagnosis, characterization, and longitudinal follow-up of cardiac tumors [3]. However, imaging was not readily available for this patient due to resource limitations. Nevertheless, definitive diagnosis remains histopathological, with characteristic features including glycogen-rich cytoplasm and the presence of *spider cells*, as observed in this case.

Autopsy findings were instrumental in this case, providing valuable insight into the extent of disease and its underlying pathophysiological mechanisms. The postmortem examination demonstrated the significant mass effect of the tumor and its contribution to pulmonary hypoplasia, thereby clarifying the cause of death. This emphasizes the continued importance of autopsy in elucidating rare pediatric conditions, particularly in resource-constrained environments.

Regional data on cardiac tumors in the Caribbean remain limited; however, studies from the University Hospital of the West Indies have documented the occurrence of primary cardiac tumors within this population [9]. This highlights the need for further regional research to better characterize the epidemiology and clinical outcomes of such cases.

## Conclusions

Cardiac rhabdomyoma, although typically benign, may result in fatal outcomes when lesions are large and associated with significant structural and functional compromise. This case highlights the potential for such tumors to contribute to pulmonary hypoplasia and rapid clinical deterioration in the neonatal period.

Early detection through antenatal imaging, coupled with appropriate postnatal evaluation and multidisciplinary management, is essential for optimizing clinical outcomes. In resource-limited settings, where advanced diagnostic modalities may be restricted, autopsy remains a vital tool for establishing the diagnosis and enhancing understanding of disease processes.
